# Complementarity in dietary supplements and foods: are supplement users vegetable eaters?

**DOI:** 10.1080/16546628.2017.1361769

**Published:** 2017-08-13

**Authors:** Hyoung-Goo Kang, Hailey Hayeon Joo, Kyong Duk Choi, Dongmin Lee, Junghoon Moon

**Affiliations:** ^a^ Hanyang University Business School, Hanyang University, Seoul, South Korea; ^b^ Department of Economics, Sogang University, Seoul, South Korea; ^c^ Food Biz Lab, Program in Regional Information, Seoul National University, Seoul, South Korea

**Keywords:** Dietary supplements, fruits and vegetables, complementarity, health consciousness, scanner data

## Abstract

**Background**: The consumption of fruits, vegetables, and dietary supplements correlate. Most previous studies have aimed to identify the determinants of supplement uses or the distinct features of supplement users; this literature lacks a discussion on dietary supplement consumption as a predictor of fruit and vegetable consumption.

**Objective**: This study examines how dietary supplement consumption correlates with fruit and vegetable consumption by combining scanner data and surveys of Korean household grocery shopping.

**Methods**: Propensity score matching (PSM) is used to identify the relationship between dietary supplement consumption and fruit and vegetable consumption in a household. A logit regression using supplement consumption as the dependent variable is used. Then, the supplement takers (the treatment group) are matched with non-takers (the control group) based on the propensity scores estimated in the logit regression. The fruit and vegetable consumption levels of the groups are then compared.

**Results**: We found that dietary supplement use is associated with higher fruit and vegetable consumption. This supports the health consciousness hypothesis based on attention bias, availability heuristics, the focusing effect, and the consumption episode effect. It rejects the health substitute hypothesis based on economic substitutes and mental accounting.

**Conclusions**: Future research on the health benefits of dietary supplements should address the complementary consumption of fruits/vegetables and their health benefits to avoid misstating the health effects of supplements.

## Introduction

Fruit and vegetable consumption is an important topic because of the health benefits and market size of these foods. This study explores whether buyers consume fruits and vegetables jointly with dietary supplements. The existing literature reports that the consumption of fruits/vegetables and dietary supplements correlate [1–4], though a consensus is lacking on the use of dietary supplements as a predictor of fruit and vegetable consumption. Most prior studies have aimed to identify the predictors of supplement use or the characteristics of supplement users [1–4]. This study poses the following research question: in reverse, does supplement use predict the consumption of vegetables and fruits?

The current literature’s omission of reverse association leaves an important gap. The dietary supplement sectors are innovative and growing, so fruit and vegetable producers should know whether such growth is a threat or opportunity. Likewise, dietary supplement producers need to account for fruit/vegetable consumption to identify the pure health benefits of the supplements and provide consumers with proper consumption guidance.

This research fills the void left by the existing literature by analyzing this reverse association. It will contribute to future research on the dietary benefits of supplements, consumption decisions regarding dietary supplements and fruits and vegetables, and policy implications for the health and related industries.

## Hypothesis development

Prior studies have found that the consumption of fruits/vegetables and dietary supplements correlate [1–4]. However, there are mixed results about the type of association between supplement use and fruit/vegetable consumption [3,5]; some studies cite complementary relationships [1–4], while others assert that supplement use is a means to compensate for an unhealthy lifestyle (i.e., a substitution; see Table 1) [5,6]. This implies two competing hypotheses: the health consciousness hypothesis and the health substitute hypothesis, which focus on complementarity and substitutions, respectively.

**Table 1.** Literature on the type of relationships between supplement use and healthy food consumption.

Type of association	Reference	Results	Subjects	Methods
Complement	[1]	Supplement user showed healthier dietary habits	6,352 individuals from Spain	Survey
	[2]	High consumption of fruit and vegetables is associated with supplement use	13,822 individuals from a UK women’s cohort study (UKWCS)	Survey
	[3]	Supplement users eat a balanced diet more than nonusers	2,575 respondents from a 2011 CRN consumer survey (US)	Survey
	[4]	Higher consumption of fruit products was the most primary individual dietary factor	2,152 individuals from the Beaver Dam Eye Study (US)	Survey
Substitution	[5]	Participants who took dietary supplements expressed greater preference for a buffet over an organic meal	82 participants from Taiwan	Experiment
	[6]	Interviewees thought that functional foods would compensate for an unhealthy lifestyle	46 individuals from Sweden	Interview

### Health consciousness hypothesis

Much of the existing literature shows that supplement users have healthier dietary habits [1,3] or consume more fruits and vegetables than non-supplement users [2,4]. This is reportedly because ‘the health-conscious attitude predominates among dietary supplement users’ [7, p.896]. These studies report that consuming dietary supplements can generate health consciousness in the minds of consumers. Enhanced health consciousness increases interest in healthy foods, leading to the complementary consumption of fruits and vegetables. The health consciousness hypothesis is related to attention bias, availability heuristics, the focusing effect, and the consumption episode effect.

Attention bias refers to repeated thinking about a subject that then influences the thinker’s perception of said subject. When attention is focused on certain attributes of a subject, it becomes easier to disregard the other attributes [8,9]. Consuming dietary supplements can prime consumers to pay more attention to health. This causes the consumers to focus on the attributes related to health and to ignore the other attributes in food. A result of this is the increased consumption of produce that customers believe to be healthy.

Availability heuristics refer to when a consumer relies on easily available information to evaluate a subject over more relevant or complete data [10]. For example, recent events and those that evoke intense emotions are ‘easily available.’ The visibility of such events can cause consumers to overestimate their frequency. Similarly, the habit of consuming dietary supplements can lead consumers to develop psychological beliefs related to the supplements (e.g., belief in the likelihood of health events). Consumers become more invested in their personal health and overrate the threat of diseases or symptoms. Thus, they consume more fruits and vegetables because they believe in their health benefits.

The focusing effect relates to attention bias. Cognitive bias occurs when consumers emphasize one aspect of a subject cognitively while underrating the other aspects, leading to erroneous predictions [11]. The most important attribute of dietary supplements is their benefit to health, at least in the minds of consumers. Thus, while consuming dietary supplements, a consumer can develop the habit of identifying and highlighting the health benefits of foods. This leads the consumer to judge foods based on their perceived health aspects while underrating other aspects. If consumers believe fruits and vegetables are good for their health, they will consume more of them.

The consumption episode effect generates the complementary. To illustrate, suppose a consumer has a goal. This consumer can ‘highlight’ and consume a set of goods with attributes in line with the goal. This consumption highlighting produces the perception of attaining the goal in the consumer’s mind. This eventually allows the consumer to experience a climax of high utility [12]. Imagine that another consumer takes dietary products to achieve a health goal. By consuming fruits and vegetables together, this consumer can construct a goal-reaching episode and generate a satisfying climax of accomplishment.

Combining the insights from cognitive biases and the consumption episode effect produces the following health consciousness hypothesis:

Hypothesis 1 (health consciousness hypothesis): *The greater the consumption of dietary supplements, the greater the consumption of fruits and vegetables.*

### Health substitute hypothesis

Substituting dietary supplement consumption for fruits and vegetables is possible as well. Dietary supplements and produce can be substituted in consumers’ minds to alleviate health concerns. Consumers may believe that certain dietary products, such as multivitamins, are as healthy as fruits and vegetables. These consumers have incentive to choose between dietary supplements and fruits/vegetables.

These consumers may also believe that dietary supplements are a means to compensate for an unhealthy lifestyle. Chiou et al. [13] explain this phenomenon with the notion of licensing. Consumers earn a perceived license to engage in an unhealthy lifestyle after engaging in health-protective behaviors.

With mental accounting [14], consumers can record their nutrient intake in separate mental accounts. They code gains and losses when consuming foods classified by nutrient types in their mental accounts. When these consumers ingest dietary supplements, they code the amount of the nutrient inflow. Hence, they can eat fewer fruits and vegetables of similar nutrient counts if there is enough nutrient stock in the mental account.

The described subjective–substitutive relationship, licensing, and mental accounting have been used to develop the following health substitute hypothesis:

Hypothesis 2 (health substitute hypothesis): *The greater the consumption of dietary supplements, the lower the consumption of fruits and vegetables.*

## Methods

We analyzed consumer panel data compiled by the Rural Development Administration (RDA) of South Korea. It included scanner data from approximately 835 households (or 2,451 family members), tracking their grocery shopping habits from December 2009 to November 2014. The consumer panels were selected via a stratified sampling method. Stratified sampling is defined as ‘a probability sampling procedure in which simple random subsamples that are more or less equal on some characteristic are drawn from within each stratum of the population’ [15]. Wives in households attached receipts to the monthly account books. The data included complete information on household demographics and grocery shopping. Of the 835 households, 508 responded to this 2015 survey on dietary supplement consumption. Appendix A summarizes the statistics from the scanner survey data. To check for potential attrition bias, a mean test was conducted first. We found that all the variables except the age dummy (household wives under 40) differed between the two groups (see Appendix B, Table B1). To solve the attrition problem, we followed the method introduced by Little and Rubin [16]. We used the inverse of survey response logit probability for each observation’s weight to run a weighted propensity score matching (PSM) model.

Nine experiments were conducted. First, we selected the top three dietary supplements in Korea: vitamins, red ginseng, and lactobacillus. These were distinct in their characteristics and health effects. This distinctiveness enabled us to check whether the causality between dietary supplements and fruits/vegetables remained robust for different supplements. Second, we analyzed three different types of consumers: families, couples, and individuals. ‘Family’ marked instances where one or more household members consumed dietary supplements. ‘Couple’ referred to a husband and wife. ‘Individual’ referred to cases in which only the wife consumed the supplements. We then analyzed how supplement consumption among these three categories affected household fruit and vegetable consumption. Consequently, we analyzed nine combinations of causality: 3 (vitamins, red ginseng, lactobacillus) *3 (family, couple, individual) using the mean, median, and regression analysis to generate 27 main results.

We used PSM to identify causality. First, we ran a logit regression using supplement consumption as the dependent variable. Second, we matched the supplement takers (the treatment group) with the non-supplement takers (the control group) based on the propensity scores estimated by the logit regression. Third, we compared fruit and vegetable consumption among these groups.

The dependent variables were coded as 1 if a household member (family, couple, individual) consumed a dietary supplement (vitamins, red ginseng, lactobacillus). ‘Education’ was used as a dummy taking 1 if a responding wife was a college graduate. ‘Income’ described monthly income in Korean Won. ‘Homemaker’ was set as a dummy taking 1 if a responding wife was a homemaker. ‘Age below 40’ and ‘age 41–55’ were dummies to indicate age. The total number of observations was 30,235.

## Results

Table 2 presents the results of the sample mean and median tests. The numbers represent fruit and vegetable shopping values per month. The ‘Treatment’ column refers to the treatment group that consumed dietary supplements, and the ‘Control’ column is the control group that did not consume supplements. Difference tests using the mean and median values were conducted. The results show that the fruit and vegetable shopping values of the treatment group were higher than those of control group.

**Table 2.** Sample mean test.

		Treatment	Control	Difference	p-value
Vitamins	Family	119,219	109,543	9,676***	.000
	Couple	118,464	107,518	10,946***	.000
	Own	115,934	104,957	10,977***	.000
Red ginseng	Family	127,293	110,760	16,533***	.000
	Couple	126,977	109,171	17,806***	.000
	Own	125,992	106,301	19,691***	.000
Lacto bacillus	Family	131,487	109,661	21,826***	.000
	Couple	138,946	106,871	32,075***	.000
	Own	124,037	103,739	20,298***	.000

Unit: Korean Won (KRW).

Significance levels: (***) 1%.

Table 3 presents the main results of the PSM. The higher the number of children in a family unit, the more the family as a whole consumed vitamins and lactobacillus (though the parents consumed less). The consumption of red ginseng, typically a for-adult supplement, decreased in all cases as the number of children increased. The results indicate that parents consumed fewer supplements as they had more children, but overall family consumption still rose due to the children’s intake. Education and income contributed to higher supplement consumption, as was observed in previous studies [17–19]. The households with homemakers consumed fewer vitamins, but more ginseng and lactobacillus. The age of the wives also affected the household consumption of dietary supplements.

**Table 3.** Results of the logit regression and PSM.

	Vitamin			Red ginseng			Lactobacillus		
	Family	Couple	Individual	Family	Couple	Individual	Family	Couple	Individual
Education	0.281***	0.270***	0.177***	0.787***	0.548***	0.370***	−0.538***	0.203***	0.259***
	(.028)	(.023)	(.023)	(.061)	(.033)	(.026)	(.044)	(.032)	(.023)
Income	0.497***	0.612***	0.498***	−0.092	0.168***	0.344***	0.789***	0.546***	0.449***
	(.027)	(.022)	(.021)	(.059)	(.032)	(.024)	(.040)	(.031)	(.021)
Number of children	0.096***	−0.099***	−0.057***	−0.255***	−0.126***	−0.205***	0.080***	−0.384***	−0.284***
	(.017)	(.014)	(.013)	(.038)	(.020)	(.015)	(.025)	(.019)	(.014)
Homemakers	−0.232***	−0.046	−0.084***	0.196***	0.381***	0.449***	0.684***	0.300***	−0.038
	(.027)	(.021)	(.020)	(.058)	(.032)	(.024)	(.040)	(.030)	(.021)
Age below 40	−0.812***	−0.067	0.249***	−0.785***	−0.472***	−1.035***	0.349***	−0.943***	−0.451***
	(.054)	(.037)	(.035)	(.101)	(.053)	(.039)	(.082)	(.055)	(.037)
Age 41–55	0.728***	0.007	0.109***	−0.125	−0.187***	−0.694***	0.899***	−0.080	0.109***
	(.048)	(.031)	(.030)	(.080)	(.044)	(.032)	(.069)	(.040)	(.030)
Constant	−2.619***	−0.857***	0.022	−3.161***	−2.093***	−0.640***	−4.010***	−1.506***	−0.308***
	(.054)	(.036)	(.034)	(.091)	(.052)	(.037)	(.082)	(.047)	(.035)
Beta	12,074.19***	6,677.62***	11,738.33***	6,462.76***	13,702.14***	14,022.94***	18,798.71***	25,042.72***	16,014.72***
Observations	27,373	29,175	30,097	18,331	25,304	28,898	24,047	26,957	29,367
R^2^	.028	.023	.015	.023	.019	.038	.048	.044	.030

Significance levels: (***) 1%.

Note: Table 3 shows the results of weighted PSM using the inverse of the logit probability of responding to the survey as each observation’s weight.

We also regressed fruit and vegetable shopping values on the treatment group dummy – the row denoted as ‘Beta’ shows dietary supplement consumption’s average treatment effect on the treated (ATT) on the shopping values of fruits and vegetables. The control group was selected from the set of households not consuming dietary supplements at each combination of 3 (family, couple, individual) *3 (vitamins, red ginseng, lactobacillus).

The results were consistent. The treatment group consumed significantly more dietary supplements than did the control group for all combinations and almost all comparisons (mean, median, and regression). This indicates that consuming dietary supplements can cause consumers to consume more fruits and vegetables. This confirms the health consciousness hypothesis over the health substitute hypothesis. An additional substitutability was also identified in those who took dietary supplements more often than was average (see details in Appendix C).

## Discussion & conclusions

The results of this study indicate that consuming dietary supplements can increase fruit and vegetable consumption. This finding supports the health consciousness hypothesis over the health substitute hypothesis. The former hypothesis draws from attention bias, availability heuristics, the focusing effect, and the consumption episode effect, whereas the latter involves economic substitutes, licensing, and mental accounting. Prior studies showed similar results regarding the complementary relationship between supplement intake and fruit and vegetable consumption [1–4]. The results of this study extend these prior findings to suggest that supplement use can be an appropriate predictor of fruit and vegetable consumption. This follows the conclusion of a previous study that emphasized the importance of taking supplement use into account when studying diets [17].

This study uses PSM to the association between dietary supplement consumption and fruit/vegetable consumption. The PSM results reported here draw from all the available socio-demographic variables of the panels in our data set. However, an uncontrolled variable may still exist that could influence the consumption of dietary supplements, fruits and vegetables, or both. In the event that this variable exists, the estimates of the present study would be biased.

This study’s findings have numerous implications. First, future research on the health benefits of dietary supplements should address the complementary consumption of fruits and vegetables. If fruits and vegetables are perceived as healthy already, this can exaggerate empirical results on the health benefits of supplements. Second, the complementarity between supplements and produce suggests that some consumer sets over- or under-consume some nutrients. Policy makers should carefully consider consumers’ complementary consumption trends. Third, producers and retailers should develop bundled or other creative goods using the information in this study on complementarity and the health consciousness hypothesis. This is especially important as a marketing implication for shelf space management.

## Appendix A. Summary statistics for scanner and survey data for dietary supplements

**Household demographics**

Data was used from 508 households (Table A1). The ‘dine-out’ dummy takes 1 if a household dines out at least two times per month. The ‘education’ dummy takes 1 if a respondent is a college graduate. The ‘age (below 40)’ dummy takes 1 if the participant is 40 years or below. The ‘age (41–55)’ dummy takes 1 if the respondent is between 41 years and 55 years. The ‘homemaker’ dummy is 1 if a respondent is a homemaker (working wife takes 0). The ‘income’ dummy takes 1 if the household’s income is more than 3.5 million KRW per month. The ‘husband’ dummy takes 1 if a husband is present in the household.

**Table A1.** Household demographics.

Variables	Average	Standard Deviation	Min	Max
Number of family members	3.82	1.045	1	9
Number of children	1.75	0.783	0	5
Dine-out dummy	0.45	0.498	0	1
Education dummy	0.36	0.482	0	1
Age	43.64	7.720	25	66
Age (below 40) dummy	0.38	0.485	0	1
Age (41–55) dummy	0.55	0.498	0	1
Homemaker dummy	0.52	0.500	0	1
Income dummy	0.37	0.485	0	1
Husband dummy	0.83	0.373	0	1

**Types and consumption of dietary supplements**

Table A2 presents the types and consumption amounts of various dietary supplements. The values indicate who in the household consumes the dietary supplements specified in the first column and to what extent. The columns ‘Wife,’ ‘Husband,’ and ‘Children’ indicate whether the wife, husband, or one of their children consume the supplements.

**Table A2.** Household demographics.

	Wife	Husband	Children
Red ginseng	28.3 %	30.7 %	20.9 %
Vitamins	64.7 %	57.8 %	56.3 %
Calcium	13.2 %	5.5 %	8.7 %
Glucosamine	3.9 %	3.3 %	0 %
Omega 3	24.6 %	22.1 %	8 %
Lactobacillus	41.3 %	24.6 %	39.2 %
Appetite suppressant	3.7 %	2.7 %	1.6 %
Protein	4.4 %	5.3 %	5.5 %
Juice	32 %	33.8 %	24.8 %
Other	14.6 %	13.3 %	5.1 %

**Purpose of consuming dietary supplements**

Table A3 lists the wife’s, husband’s, and children’s reasons for consuming dietary supplements in the household.

**Table A3.** Purpose of consuming dietary supplements.

	Wife	Husband	Children
Nourishing supplement	20.8 %	16.8 %	29.2 %
Physical strength	14.6 %	19.3 %	26.3 %
Disease prevention	22.9 %	26 %	20.2 %
Weight control and diet	4.4 %	1.9 %	4.8 %
Fatigue recovery	27.3 %	32.4 %	14.1 %
Other	2.5 %	2.2 %	3.5 %

## Appendix B. Attrition issue

A mean test was conducted to check for differences in the descriptive statistics of the panels’ observable characteristics according to survey responses (i.e., ‘Response’ or ‘No Response’). The results of this test indicated that all the variables except the age dummy (age below 40) varied between the two groups (see Table B1).

To solve the attrition problem, we followed the method introduced by Little and Rubin [16]. We used the inverse of the survey response logit probability as each observation’s weight to run a weighted PSM model. Although the characteristics among those who did and did not respond to the survey were significantly different (as demonstrated by the marginal effects shown in Table B1), the weighted PSM results were not qualitatively different from those without weights.

**Table B1.** Response statuses.

	Mean Test			
	Response	No response	Difference	Marginal effect
Education	0.372	0.335	0.037***	0.017*** (.002)
Income	0.488	0.410	0.078***	0.053 (.002)
Number of children	1.741	1.773	−0.032***	−0.019*** (.001)
Homemakers	0.523	0.595	−0.072***	−0.052*** (.002)
Age below 40	0.241	0.250	−0.009	0.048*** (.003)
Age 41–55	0.617	0.575	0.042***	0.049*** (.003)

Significance levels: (***) 1%

## Appendix C. Frequency of dietary supplement intake

A household’s pattern of fruit and vegetable consumption relates to how often its members take dietary supplements. In the present survey, households reported how often they consumed any dietary supplement rather than reporting on individualize items. As such, PSM scores reflected the households’ overall consumption frequencies for dietary supplements.

These results are presented in Table C1. For participants who consumed any dietary supplements, the dummy variable took 1 if the frequency of supplement use was greater than or equal to the mean frequency (e.g., 3.93 times per week for families, 4.30 for couples, and 4.59 for individuals), and 0 if less. The results were consistent in showing that those who took dietary supplements more often than the average also spent more on fruits and vegetables by approximately 6,500–11,700 KRW when grocery shopping.

**Table C1.** PSM for frequent users.

	Dummy for Frequent User		
	Family	Couple	Individual
Education	0.199*** (.023)	0.081*** (.023)	0.179*** (.024)
Income	0.480*** (.021)	0.517*** (.021)	0.089*** (.022)
Number of children	0.059*** (.014)	0.008 (.014)	−0.074*** (.015)
Homemakers	−0.036 (.021)	−0.047 (.021)	−0.392*** (.022)
Age below 40	−0.268*** (.037)	−0.256*** (.037)	−0.424*** (.038)
Age 41–55	−0.085*** (.031)	−0.292*** (.031)	−0.261*** (.033)
Constant	−0.274*** (.036)	−0.121*** (.036)	0.639*** (.039)
Regression (beta)	6,478.883***	5,708.120***	11,724.431***
Observations	27,469	27,315	25,529
R^2^	.015	.014	.012

Significance levels: (***) 1%

PSM is based upon the frequency of taking any dietary supplements.
